# A proof of concept for wear/non-wear classification using accelerometer data in daily activity recording: Synthetic algorithm leveraging probability and continuity of zero counts

**DOI:** 10.1371/journal.pone.0309917

**Published:** 2024-10-22

**Authors:** Natsumi Nishiyama, Shoji Konda, Issei Ogasawara, Ken Nakata

**Affiliations:** 1 Department of Health and Sport Sciences, Graduate School of Medicine, Osaka University, Toyonaka, Osaka, Japan; 2 Department of Sports Medical Biomechanics, Graduate School of Medicine, Osaka University, Suita, Osaka, Japan; Hamasaki Clinic, JAPAN

## Abstract

Wearable devices are increasingly utilized to monitor physical activity and sedentary behaviors. Accurately determining wear/non-wear time is complicated by zero counts, where the acceleration-based indexes do not estimate activity intensity, often leading to misclassifications. We propose a novel synthetic classification algorithm that leverages both the probability and continuity of zero counts, aiming to enhance the accuracy of activity estimation. The physical activity data were obtained from 12 office workers wearing wearable devices with 3-axis accelerometers. The wear/non-wear times are classified by the commonly used current method (zero counts lasting longer than 60 minutes are classified as non-wear) and the proposed method. In the proposed method, only times that satisfy the following two criteria are classified as the wear time. (1) The appearance probability preceding and following 60 minutes must be less than the threshold value. (2) The number of consecutive zeros must be less than 10 minutes. The effectiveness of both the current and proposed classification methods was evaluated against the actual behavioral records. This evaluation utilized simulation-based augmented data, which was implemented to address the limited variability inherent in the original dataset. The range of recall, specificity, precisions, and accuracy classified by the current method were 0.93–1.00, 0.93–0.96, 0.85–0.88, and 0.94–0.97, respectively. Indeed, the proposed method shows 0.95–1.00, 0.99–1.00, 0.97–1.00, and 0.98–1.00, respectively. The reduction of misclassification of non-wear time as wear time was achieved by the synthetic classification algorithm. The performance of the proposed approach showed accurate classification of the wear/non-wear time of wearable sensors in office workers.

## Introduction

Habitual physical activity has been recommended for various benefits, including prevention of obesity, hypertension, diabetes, cardiovascular disease, and depression and reduction of anxiety [1, 2]. World Health Organization (WHO) has recommended 150–300 minutes of moderate-intensity physical activity (3.0 to 5.9 METs: Metabolic Equivalent for Tasks) per week or 75–150 minutes of vigorous-intensity physical activity (more than 6.0 METs) per week, based on the association between the incidence of non-communicable diseases such as diabetes, cancer, and cardiovascular disease [2]. Interestingly, health problems are associated with prolonged sedentary behavior, independent of moderate to vigorous physical activities [3–6]. The sedentary behavior has been defined as all arousal behaviors with an energy expenditure of 1.5 METs or less in the sitting or lying position [7]. In the general adult, approximately 55–60% of waking hours are spent in sedentary activities [3]. Adults with more than 7 hours of sedentary time per day have a risk of cardiovascular disease and mortality, even if the recommended physical activity by WHO is met [5, 8, 9]. Brief periods (e.g., within 2–3 minutes) of light-intensity physical activity (1.6 to 2.9 METs) during sedentary behavior (sedentary break) are beneficial for the reduction of waist circumference, body mass index, triglycerides, 2-hour blood glucose levels, and blood pressure [10, 11].

Questionnaire surveys are advantageous for evaluating the total physical activity in a large population study while not evaluating the intensity, short activity time (shorter than 15 minutes), and frequency of activity accurately [12, 13]. Wearable devices with built-in accelerometers have been widely used to assess physical activity and sedentary daily living activities [11, 14–18]. The commercially available wearable devices calculate summary activity intensity variables such as “counts” and “METs” from the raw acceleration signal [19–21]. These summary variables are more understandable than the acceleration signal for the users. In addition, it allows for memory savings by reducing the temporal resolution of raw acceleration signal (over 30Hz) to longer intervals [21], such as ten seconds or a minute, making it more efficient for conserving storage space in long-term monitoring. However, the wear/non-wear time classification of wearable devices has a significant limitation. A cause of misclassification is when the devices did not record activity data despite being wear time, which frequently appears during sedentary behavior [22]. This misclassification probably arises when the raw acceleration signal during a specific interval fails to surpass a defined threshold, causing zero value of summary variables (0 counts or 0 METs), called “zero counts”. The misclassification causes an underestimation of total sedentary time and misrecognition of short intermittent sedentary behavior, even if it is long and continuous. Another cause of misclassification is the period that the devices record activity data, even if non-wearing the device due to artificial noises (e.g., accidental movement by hand) [22]. The artificial noises cause the overestimation of the total physical activity time or sedentary behavior. Thus, the accelerometry data may not reflect the physical activity and sedentary behavior in free-living conditions.

Various data processing algorithms for classifying wear/non-wear time have been developed for raw accelerometry data and processed summary variables (counts or METs). If raw accelerometry data can be stored in the device, the rich information, which has a three-dimensional signal with a high time resolution, can be used for classifying wear/non-wear time with machine learning and neural network techniques, skin temperature, and electrocardiogram [23–26]. If the processed summary variables (counts or METs) are only stored in the device, one-dimensional data with low time resolution can be used [23]. Even today, many devices store only summary variables, and processing for such signals is still necessary.

The zero counts of summary variables are likely to appear continuously during the actual non-wear time, while zero counts are likely to occur intermittently during wear time [22]. The most common threshold is that only continuous zero counts of 60 minutes or more are considered a non-wear time for adults [15, 22, 27–29]. The threshold has been recommended to change for children and older adults because the duration of sedentary behavior differs among these groups [30, 31]. However, with a threshold of a long-time window, such as 60 or 90 minutes, the non-wear time below the threshold is considered wear time. Furthermore, the artificial noise due to accidental movement during non-wear time is also classified as wear time [30]. Therefore, the short non-wear time below the threshold cannot be classified correctly, which is expected to affect the total time, duration, and frequency of sedentary behavior. On the other hand, if the threshold is shortened, frequent and consecutive zero counts may not be adequately corrected as wear time. Therefore, we hypothesized that it would be difficult to solve this problem solely using the duration-based approach with a continuous number of zero counts. This study aimed to propose a novel approach for solving this problem: a synthetic classification algorithm of wear/non-wear time leveraging probability and continuity of zero counts.

We propose a novel approach, aiming to classify wear and non-wear time more accurately by leveraging the probability and continuity of zero counts, particularly with the HJA-750C Active Style Pro, a research-grade activity tracker equipped with a 3-axis accelerometer on the waist. This device, which calculates estimated METs based on recorded 3-axis acceleration, has been validated through the Douglas bag method [21, 32, 33]. Although many studies have utilized the HJA-750C Active Style Pro, employing a simple duration-based classification where zero counts lasting longer than a generally accepted threshold (usually 60 minutes) are deemed non-wear [34–38], we seek to refine this process. By focusing on this specific device, our research intends to validate the effectiveness of our innovative method, which incorporates considerations of zero count probability and continuity to distinguish between wear and non-wear times more accurately.

## Methods

### Participants and data collection

Twelve office workers (three males and nine females, mean age 33.2±10.9 years) were included in the study. This study was approved by the Ethical Review Board Osaka University Hospital (Approval No. 19537), and informed consent for participation was obtained from the participants before the data collection. This study was conducted in accordance with the Declaration of Helsinki. The participants wore a wearable device (HJA-750C Active Style Pro, OMRON HEALTHCARE Co., Ltd., Kyoto, Japan). The participants were recruited from April 12^th^ 2022 to July 29^th^ 2022. The device outputs the “0 METs” as the indicator of non-wear time, such as the “zero counts” in another device, and we called “0 METs” as “zero counts” in this study. The time series data of physical activity intensity (METs) were obtained at one-minute intervals during working hours (approximately 7 hours). During the approximately 7-hour measurement period, a 45 minutes non-wear period and 10 or 20 minutes non-wear period were experimentally set up, and participants were instructed to non-wear the wearable device. The commonly used criterion for classifying non-wear time is that the zero counts lasing longer than 60 minutes. In this threshold, the non-wear times of less than 60 minutes was misclassified as wear times. Therefore, it was crucial to accurately classify non-wear times of less than 60 minutes, and the non-wear time being set at maximally 45 minutes in this study. Each participant also recorded the start and end time of non-wear in one-minute increments. Twelve participant’s data were split into the development (eight participants) and validation (four participants) data sets.

### Proposed method

We propose a synthetic classification algorithm of wear/non-wear time based on the probability of the record of zero counts and the consecutive number of zero counts, named the “SynProC algorithm” (**Syn**thesize **Pro**bability and **C**ontinuity of zero counts).

#### Wear/non-wear classification utilizing the probability of recorded zero counts

**Fig 1:**
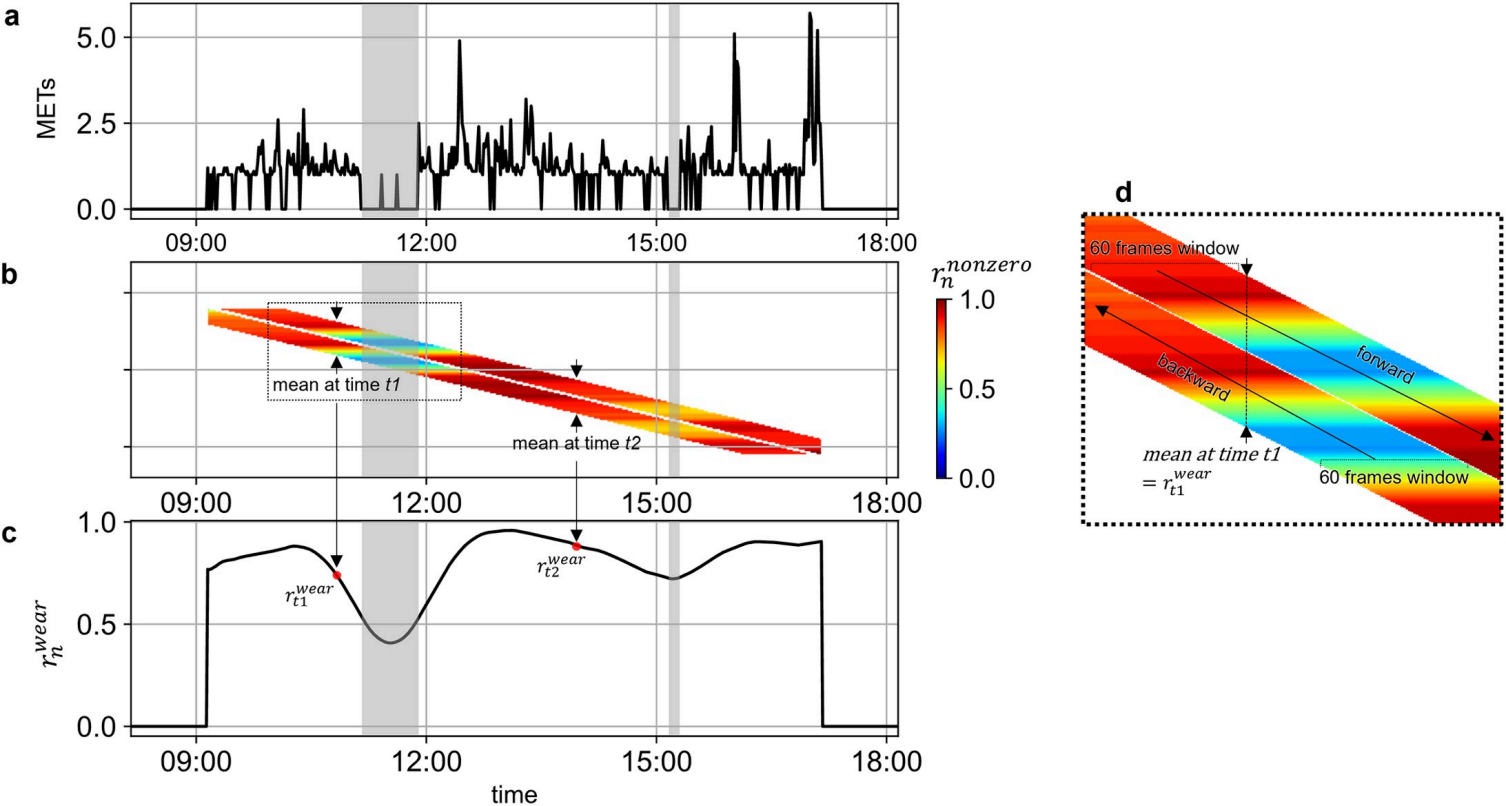
Process of calculating the wear probability (${\text{r}}_{\text{n}}^{\text{wear}}$). (a) original data recorded by the wearable device (black line) and non-wear time reported by the behavioral record (gray bands) (b) the probability of the nonzero value ($r_{n}^{nonzero}$) appearing in each 60 minutes time window during the forward and backward 60 minutes sliding windows (c) the wear probability ($r_{n}^{wear}$) at each time was calculated as the mean value of $r_{n}^{nonzero}$ at each time.

Fig 1a shows the time-series activity intensity data acquired by the wearable device. From this initial time to the end, the time window was moved forward one minute at a time. This sliding window method enabled the continuous calculation of the probability that a nonzero value appears in each 60 minutes window. For each window position, the number of times a nonzero value appeared was calculated and defined as $r_{n}^{nonzero}$ for the times within that time window (Fig 1b and 1d). The integer *n* ranges from 1 to 1440, representing the total number of 60 minutes periods in a day (60 minutes × 24 hours). Since the number of $r_{n}^{nonzero}$ decreases for the last 60 minutes, the same processing was applied in the opposite direction from the end time to the initial time (Fig 1b and 1d). Finally, the mean value of each time was calculated and defined as a wear probability ($r_{n}^{wear}$) of each time (Fig 1c). Based on the time-series changes in the wear-probability ($r_{n}^{wear}$), the intervals that exceed a certain threshold (*T*^*p*^) are classified as wear, and the intervals below the threshold are classified as non-wear (1).


Lpwear=1:wear(rnwear≥Tp)0:nonwear(rnwear<Tp) (1)

**Fig 2:**
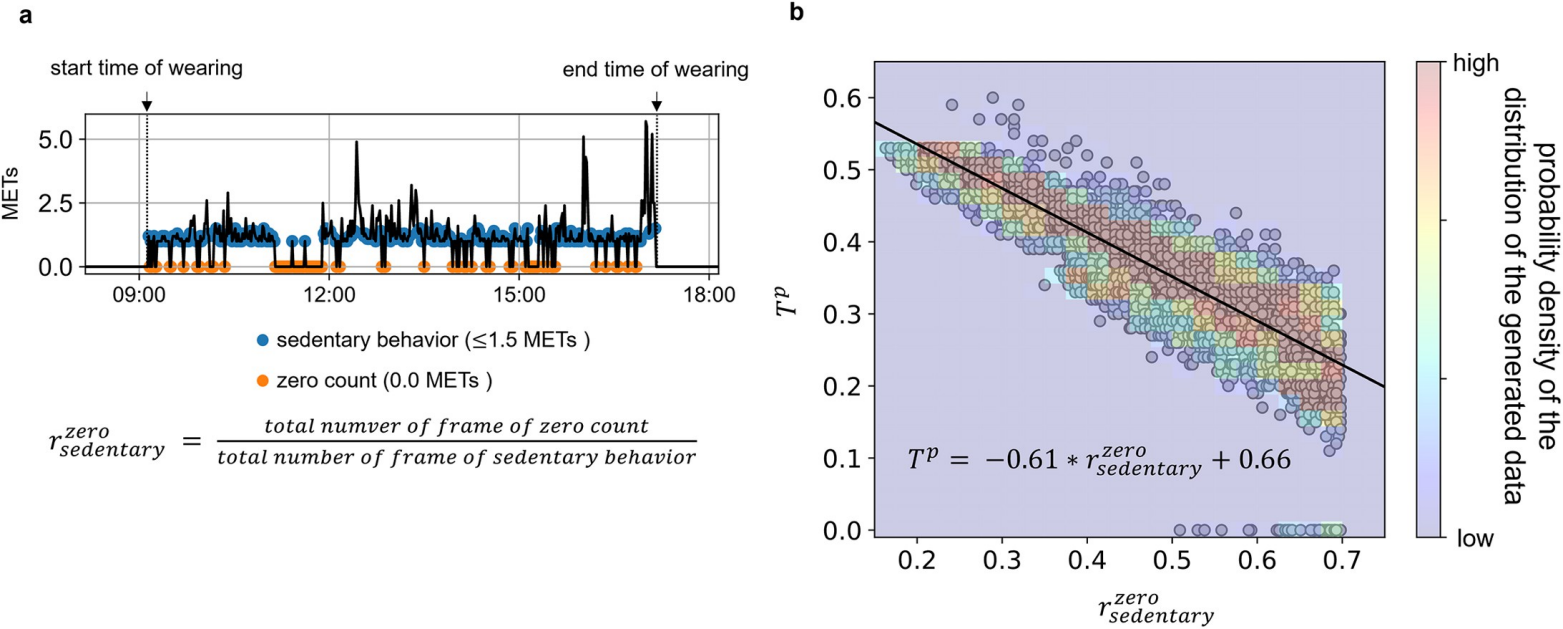
(a) The probability of the appearance of zero counts relative to the total time of sedentary behavior ($r_{sedentary}^{zero}$) was calculated by the total time for each zero count and sedentary behavior. (b) The optimal threshold was estimated as the function of $r_{sedentary}^{zero}$. Scatters show the relationship between the $r_{sedentary}^{zero}$ and the optimal threshold, and the color map shows the probability of the distribution of the generated data by the simulation for data augmentation.

An optimal threshold (*T*^*p*^) must be set to classify wear/non-wear. We found that the optimal threshold (*T*^*p*^) that most closely matches the behavioral record of wear/non-wear time recorded by each participant was able to estimate by a linear regression model of the probability of record of zero counts relative to the total time of sedentary behavior ($r_{sedentary}^{zero}$) (Fig 2a). As a result, it was confirmed that $r_{sedentary}^{zero}$ was distributed in the 16–69% range for 7 hours of record obtained from 8 office workers, demonstrating variability of the pattern of appearance of zero count even in the homogeneous office workers who were all engaged in similar desk-based work. However, we thought it would be difficult to verify whether the optimal threshold value differs depending on the pattern of zero counts even for the same occurrence rate, using only the original data recorded by the eight office workers. Therefore, we conducted a simulation-based data augmentation in which $r_{sedentary}^{zero}$ was gradually increased from the original data of eight participants to overcome the issue of limited data diversity due to a small number of participants. This process artificially broadened the dataset’s diversity, allowing us to validate our algorithm against a broader range of zero counts occurrences. The simulation generated zero counts at random times depending on the target $r_{sedentary}^{zero}$ during the sedentary behavior (S1 Appendix). Ten simulations were performed for each target value of $r_{sedentary}^{zero}$, and generated 4220 patterns from the original data of eight individuals, resulting in various patterns of zero counts occurrence (total 4228 patterns). Furthermore, a linear regression model for estimating optimal threshold (*T*^*p*^) from $r_{sedentary}^{zero}$ was developed by a robust regression (Huber regression) that accounts for the effect of outliers and the obtained prediction equation shown below (Fig 2b) (2). When this regression model is applied to multiple days of data for several participants, *T*^*p*^ is calculated for each participant’s daily data.


$$T^{p}=-0.61*r_{sedentary}^{zero}+0.66$$ (2)

#### Wear/Non-wear classification utilizing the continuity of zero counts

**Fig 3:**
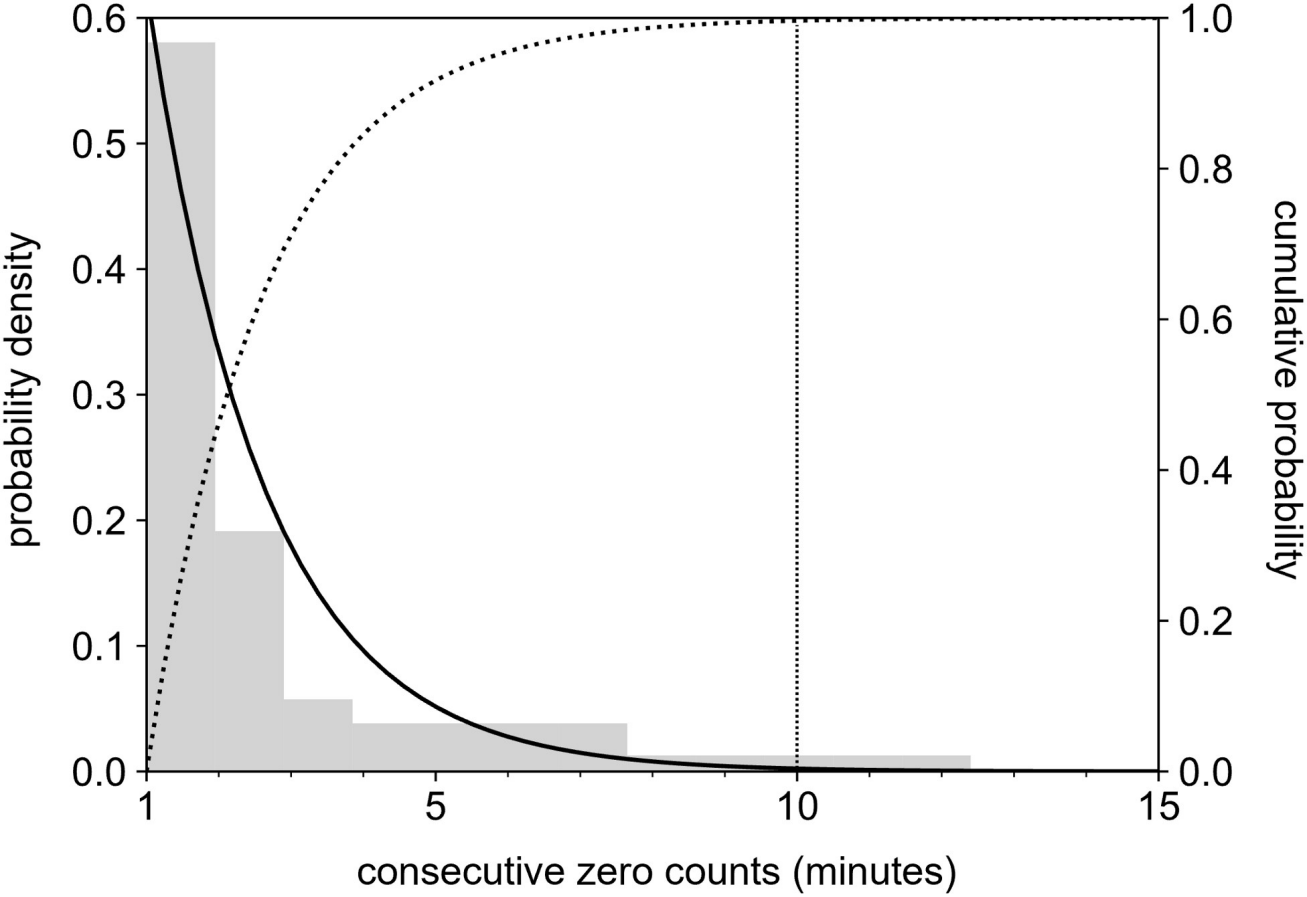
The histogram of consecutive zero counts (gray bar), probability density function (solid line), and cumulative probability function (dotted line) during the wear time.

This classification is intended to detect the short non-wear times and timing of changes between wear and non-wear. The estimation was based on the feature that zero counts are consecutive during non-wear times, while zero counts appear intermittent during wear times (Fig 3). Therefore, the time included in the interval with consecutive zero counts ($C_{n}^{zero}$) above a certain threshold (*T*^*c*^) was estimated as non-wear times. In contrast, the time included in the interval with consecutive zero counts ($C_{n}^{zero}$) below the threshold (*T*^*c*^) was estimated to be classified as wear times.


Lcwear=1:wearCnzero≤Tc0:nonwear(Cnzero>Tc) (3)

We found that consecutive zero counts up to 10 minutes could occur during the wear time for eight participants, so the threshold (*T*^*c*^) was set at 10 minutes in this study (Fig 3).

#### Synthesis of two classifications and correction of zero counts

When both the labels ($L_{p}^{wear},$ $L_{c}^{wear}$) indicate wear ($L_{p}^{wear}=1\cap L_{c}^{wear}=1$), the time that shows zero counts were reclassified as wear time ($L_{s}^{wear}=1$), the activity intensity of reclassified time was corrected by substituting 1.0, which is the minimum value of activity intensity classified as sedentary behavior.

### Validation

Excluding the data of eight participants used as training data, the data from the remaining four participants were used for cross-validation. We conducted a simulation to augment the variety of data (as described in Method and S1 Appendix) from the original data of four participants. The proposed method was applied to the obtained 2054 patterns of time-series data, and the estimated classification results of wear/non-wear were obtained for each time. As a comparison, classification using the original data without any data correction (classification as wear if the original data was 1.0 METs or higher) and the generally used current method. As the current method, we used the definition of zero counts lasting longer than 60 minutes, which is classified as non-wear. It was used as a comparison because the wear/non-wear time classification has been widely used in the study employing the wearable device (HJA-750C Active Style Pro) [34–38]. The classification performance was verified by comparing the estimated classification results of each original data, the current method, and the proposed method with the actual wear/non-wear behavior records for each time. A contingency table consisting of the actual wear/non-wear and the estimated classification result (wear/non-wear) was created (Table 1), and the performances were calculated from the following Eqs (4)–(7).


$$recall=\frac{TP}{TP+FN}$$ (4)

$$specificity=\frac{TN}{TN+FP}$$ (5)

$$precision=\frac{TP}{TP+FP}$$ (6)

$$accuracy=\frac{TP+FP}{TP+FP+TN+FN}$$ (7)

**Table 1:** Contingency table of wear/non-wear classification.

	Actual behavior (behavior report)	
Estimated classification	Wear	Non-wear
Wear	TP	FP
Non-wear	FN	TN

TP: True Positive, FP: False Positive, FN: False Negative, TN: True Negative

## Results

**Fig 4:**
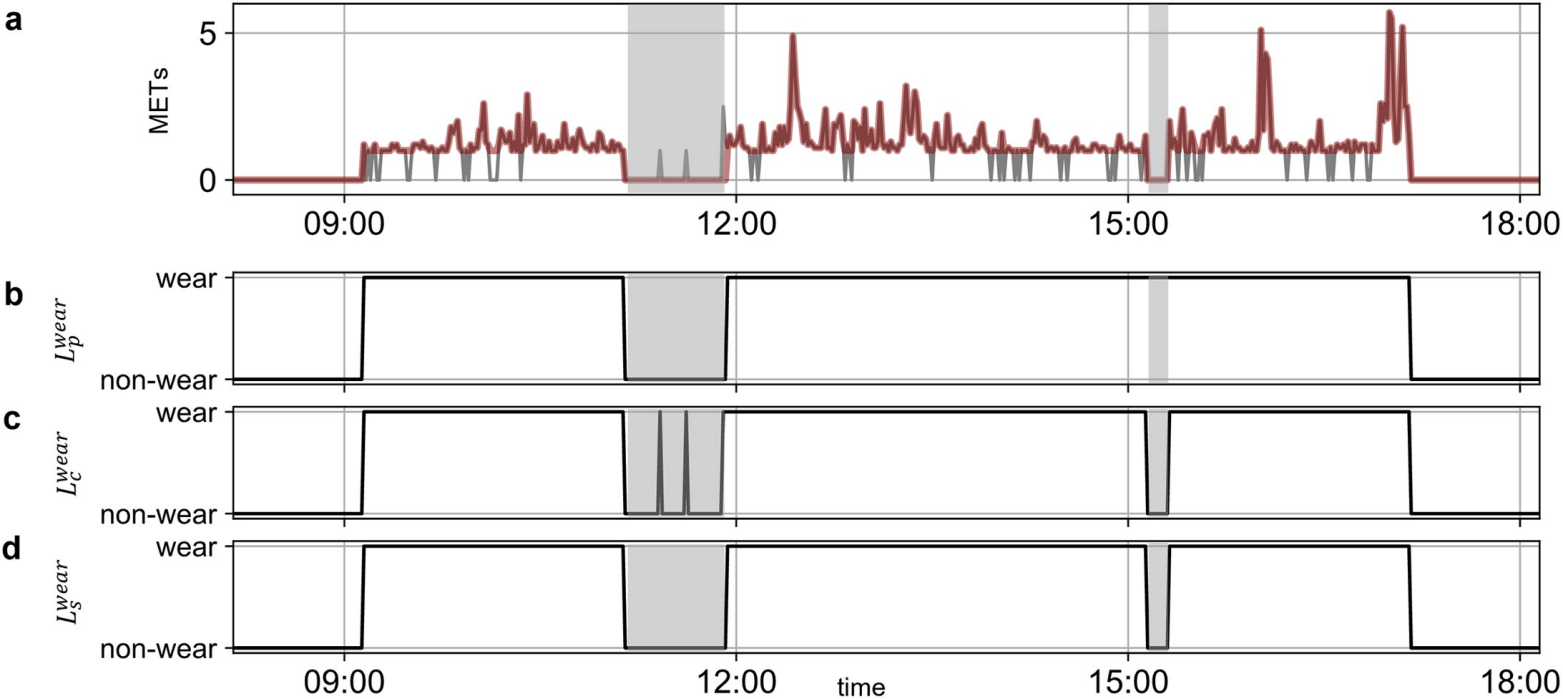
(a) original data (gray) and corrected data by the proposed method (red). Gray bands indicate the non-wear time recorded by the behavioral record. When both the labels ($L_{p}^{wear},$ $L_{c}^{wear}$) indicate wear (b and c), the time that shows zero counts were re-classified as wear time ($L_{s}^{wear}$) (d).

Fig 4 shows the typical example of the original data, corrected data by the current method, and corrected data by the proposed method. The original data showed zero counts during the sedentary behavior and the artificial noise during the non-wear time. The current method corrected the zero counts, but the non-wear time of less than 60 minutes was also misclassified as the wear time. Indeed, the proposed method corrected the zero counts and accurately classified the non-wear time.

**Fig 5:**
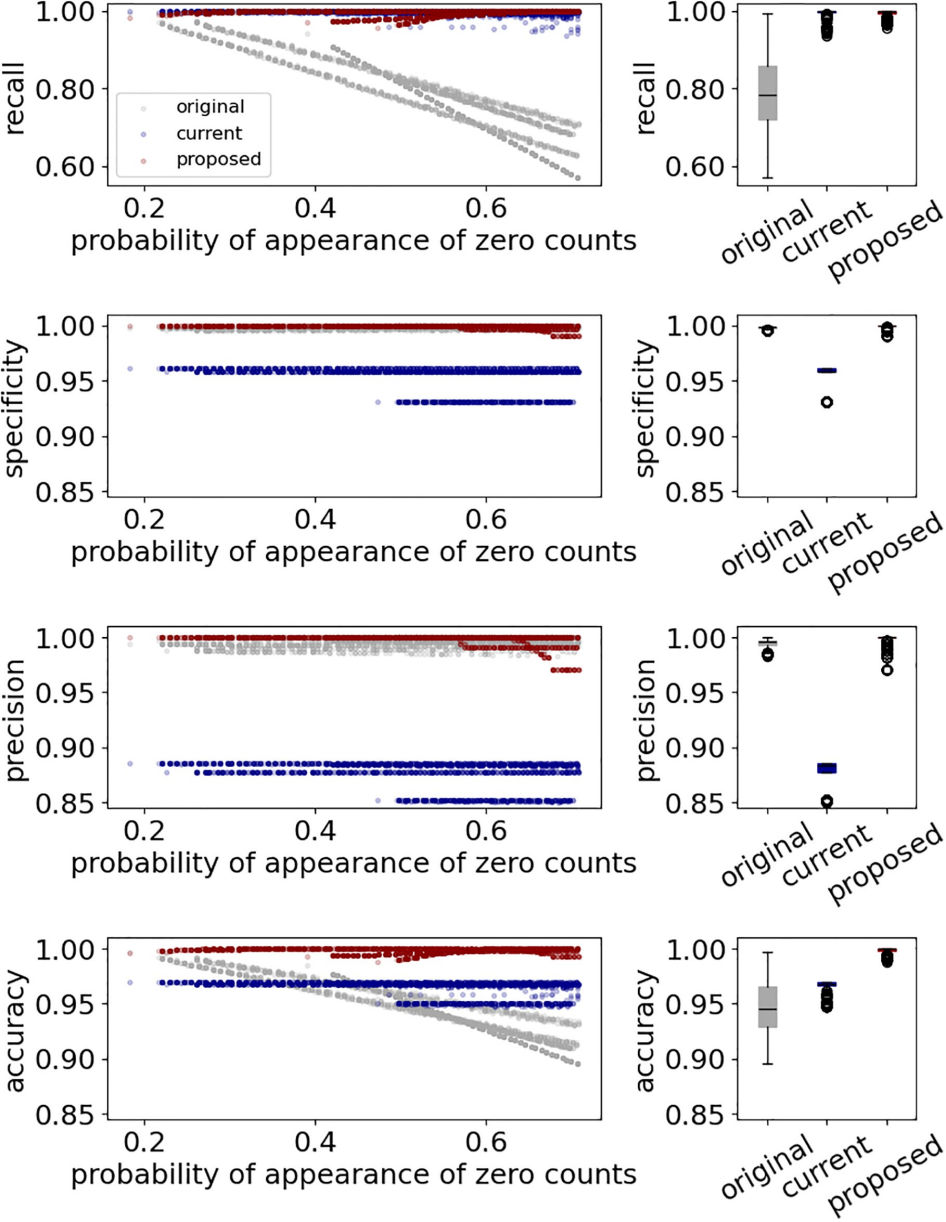
Performance (recall, specificity, precision, and accuracy) of the original, current, and proposed methods. The left panels show the relationship between the probability of the appearance of zero counts and the performance index. The right panels show the box plot of the original, current, and proposed method.

Fig 5 shows the recall, specificity, precision, and accuracy of 2054 patterns, classifying the wear/non-wear time by the original, current, and proposed methods, respectively. The median recall, specificity, precisions, and accuracy classified on the original data were 0.78 (min. 0.56 –max. 0.99), 0.99 (min. 0.99 –max. 1.00), 0.99 (min 0.98 –max. 1.00), and 0.94 (min 0.89 –max 0.99), respectively. The accuracy, recall, specificity, and precision tended to decrease as the probability of the record of zero counts increased. The median recall, specificity, precisions, and accuracy classified on the corrected data by the current method were 1.00 (min. 0.93 –max. 1.00), 0.95 (min. 0.93 –max. 0.96), 0.88 (min. 0.85 –max. 0.88), and 0.96 (min. 0.94 –max. 0.97), respectively. Indeed, the median recall, specificity, precisions, and accuracy classified on the corrected data by the proposed method were 1.00 (min. 0.95 –max. 1.00), 1.00 (min. 0.99 –max. 1.00), 1.00 (min. 0.97 –max. 1.00), and 1.00 (min. 0.98 –max. 1.00), respectively.

## Discussion

We proposed the concept of the novel synthetic approach leveraging probability and continuity of zero counts to enhance wear/non-wear classification using accelerometer data for daily activity recording. The recall was sufficiently high in both the current and proposed method. In contrast, the specificity, precision, and accuracy of the proposed method were higher than those of the current method—the reduction of misclassification of non-wear time as wear time derived from the improvement of three measures. The reduction of misclassification of non-wear time as wear time was improved by the synthetic classification algorithm of wear/non-wear time leveraging probability and continuity of zero counts. The performance of the proposed method shows that it can accurately classify the wear/non-wear time of wearable sensors in office workers whose behavior is primarily sedentary. We used the simulation-based data augmentation serving as a crucial means to address the limitations of small and homogeneous samples who were office workers all engaged in similar desk-based work. Although this approach was designed to verify the effectiveness of our algorithm, the generalizations of the results may need to be interpreted with caution.

The proposed method showed higher recall, specificity, precision, and accuracy, while the current method showed the same level of recall and lower specificity, precision, and accuracy. The current method has been reported to misclassify the actual non-wear time less than 60 minutes as wear time and falsely classify non-wear time as wear time due to artificial noises during the non-wear time (e.g., touching, shifting) [22]. The appearance of zero counts during wear time has traditionally been a major problem, and the current method is intended to detect misclassified zero counts during wear time for restoring the zero count to wear time [22]. Therefore, the recall of the current method was sufficiently high. On the other hand, the specificity, precision, and accuracy were lower than the proposed method because the due to the non-wear time less than threshold was misclassified as the wear time. If the current method were used with a shorter threshold than 60 minutes to reduce the misclassification of non-wear time as wear time, the zero counts during sedentary behavior would be misclassified as non-wear time [14], resulting in the higher specificity but the lower recall due to the insufficient restoring the zero count to wear time. Therefore, the threshold of the current method must be set by balancing recall and specificity, and it is challenging to set both recall and specificity high [31]. Our proposed method attempted to solve this problem by using the long interval (60 minutes) to calculate the wear probability, rather than using the long interval (60 minutes) to detect consecutive zero counts. The wear probability was higher when wearing the device, even if zero counts appeared in the sitting behavior. Meanwhile, the wear probability was lower when not wearing the device, even if the device contained spikes of artificial noise. The wear/non-wear classification based on the probability could extract the features of wear/non-wear time.

The threshold of consecutive zero counts was set at short (10 minutes) based on the distribution of the possible consecutive zero counts during the sedentary behavior. The 10 minutes threshold is consistent with the minimum value of the consecutive number of zero count proposed in the previous studies [22, 27]. The short threshold for the consecutive zero counts has the advantage for the accurate classification of short non-wear time. However, the short threshold of consecutive zero counts is not suitable for restoring the zero counts to the wear time, and the threshold has been generally set at 60 minutes or more [15, 22, 28, 29]. Our proposed method enabled the short threshold (10 minutes) of consecutive zero counts through the synthetic approach with the classification based on the wear probability.

We pre-defied the time window for calculating wear probability *($r_{n}^{wear}$)* and threshold of consecutive zero count (*T*^*c*^) as 60 minutes and 10 minutes, respectively. Under pre-defied these setting, the only parameter adjusted to fit each participant’s daily data is the optimal threshold (*T*^*p*^) for the wear probability *($r_{n}^{wear}$)*, which is estimated automatically from the probability of record of zero counts relative to the total time of sedentary behavior ($r_{sedentary}^{zero}$) for the total time of sitting behavior using the regression model. In contrast, when using the current method, the threshold of time interval of the consecutive zero counts has been recommended to adjust depending on the target populations [22]. In the proposed method, the higher $r_{sedentary}^{zero}$ results in the lower *T*^*p*^, increasing the misclassification of the spikes of artificial noise as wear time even if the non-wear time. We assumed that the range of $r_{sedentary}^{zero}$ is maximally 0.70, which is approximately equal to the maximum value observed in the eight participants (0.68). If $r_{sedentary}^{zero}$ exceeds 0.7 in the data, users should be aware of the loss of performance.

**Fig 6:**
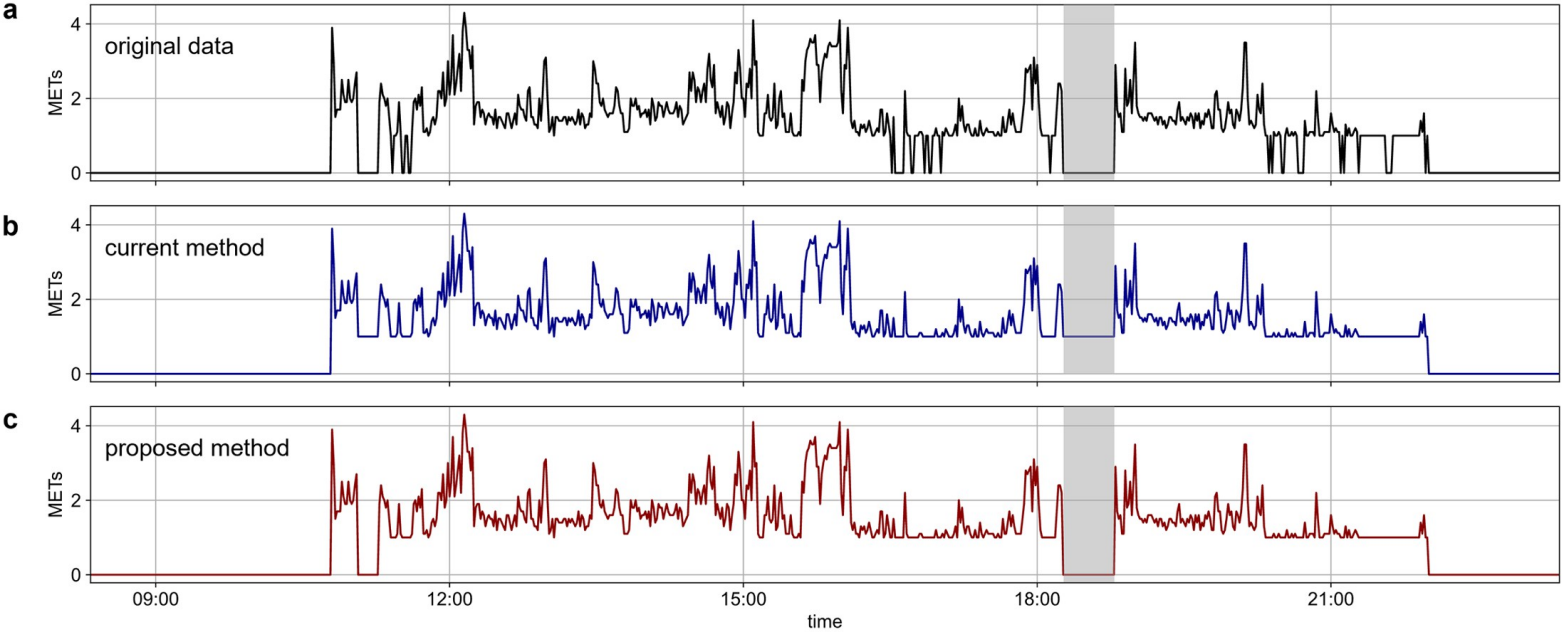
(a) The original data was recorded under the free-living conditions in a pregnant woman, (b) the data corrected by the current method, and (c) the data corrected by the proposed method. The gray bands indicate the non-wear time recorded by the behavioral record. In the current method, the non-wear time up to 60 minutes was misclassified as the wear time and corrected as the sedentary behavior (b). Indeed, the proposed method classified the actual non-wear time correctly (c). However, the misclassification was observed before 12:00 due to the over 10 minutes of consecutive zero counts (c).

The feasibility of the proposed method was tested on data recorded under the free-living condition. The wearable device recorded the physical activity data, and a behavioral record of wear/non-wear time was acquired by a pregnant woman who was 37 weeks pregnant because the pregnant woman has been generally assumed to spend more time in the sedentary behavior than adults (Fig 6). The original data underestimated the total sedentary behavior time by 48 minutes relative to the behavioral record (Fig 6a). The current method that the non-wear time up to 60 minutes was classified as wear time overestimated by 32 minutes relative to the behavioral record (Fig 6b). Indeed, the proposed method underestimated the total time of sedentary behavior by 14 minutes relative to the behavioral record (Fig 6c). A misclassification of sedentary time that could not be corrected as wearing time resulted in an underestimate of 14 minutes (before 12:00). The participant reported that she relaxed in a reclining position on the couch during this duration. Thus, the zero counts were entirely consecutive during this duration, as if the duration was non-wear time. Overall, the proposed method can reflect the actual wear/non-wear time well, suggesting that the proposed method is feasible for correcting the data under free-living conditions.

The limitations of this study include, first, the generalization performance of the proposed method, which has yet to be tested on data obtained by other wearable devices. The wearable device used in this study outputs estimated METs in one-minute epoch length from data measured by the internal tri-axial accelerometer. Although the generalization performance of the proposed method to data output at different epoch lengths and data measured by other wearable devices (models and location) needs to be verified in future research, the misclassification problem has been commonly reported in studies using various wearable devices. The parameters (time window, threshold, etc.) may need to be adjusted for each recording condition. However, the proposed synthetic approach can apply to other epoch-length and wearable devices, enhancing the wear/non-wear classification. Second, we assigned 1.0 METs for the minimum value for sedentary behavior (1.0 to 1.5 METs) to the time classified as wear time by the proposed method. The assignment of minimum intensity may underestimate the activity intensity during sedentary behavior. However, the record of zero counts is likely to be more common in sedentary behaviors, especially those that do not include low-intensity physical activity and have low energy expenditure. For example, the data on free-living conditions shown in Fig 6 showed that zero counts frequently appeared during the evening, and the behavioral record reported that the participant was relaxing on the sofa. Therefore, the effect of substituting 1.0 METs, the minimum value for sedentary behavior, for the correction of zero counts would be appropriate. Third, the present study only focused on the work hours of twelve office workers who were all engaged in similar desk-based work. After conducting the data augmentation simulation to increase the variety patterns of appearance of zero counts, the thresholds for the probability and continuity of zero counts were determined based on the original and augmented dataset. However, due to the small sample size of the original data, the uniformity of the dataset cannot be dismissed. It is crucial to test the generalizability of the proposed method and thresholds with a more diverse and larger sample in future studies.

## Conclusion

We proposed the concept of the novel zero counts data processing approach that more closely resembles actual activity (wear/non-wear) to accurately evaluate the duration and frequency of physical activity and sedentary behavior. The misclassification of non-wear time as wear time was decreased by the synthetic classification algorithm of wear/non-wear time leveraging probability and continuity of zero counts. The performance of the proposed method shows that it can accurately estimate sensor wear/non-wear in office workers whose behavior is primarily sedentary. Therefore, the proposed approach is expected to apply to data sets recorded from participants who are expected to engage in sedentary behavior, such as older adults and patients.

## Supporting information

S1 Appendix Simulation-based data augmentation. (DOCX)

S1 Data Data for defining optimum threshold. (XLSX)

S2 Data Data for validation. (XLSX)

S3 Data Data of pregnant woman. (CSV)
