# Supplementary material for: A proof of concept for wear/non-wear classification using accelerometer data in daily activity recording: Synthetic algorithm leveraging probability and continuity of zero counts
Source: PLoS One. 2024 Oct 22;19(10):e0309917. doi: 10.1371/journal.pone.0309917 (PMC11495570; doi:10.1371/journal.pone.0309917)
Supplement: S1 Appendix — (DOCX) [file pone.0309917.s001.docx]

S1 Appendix


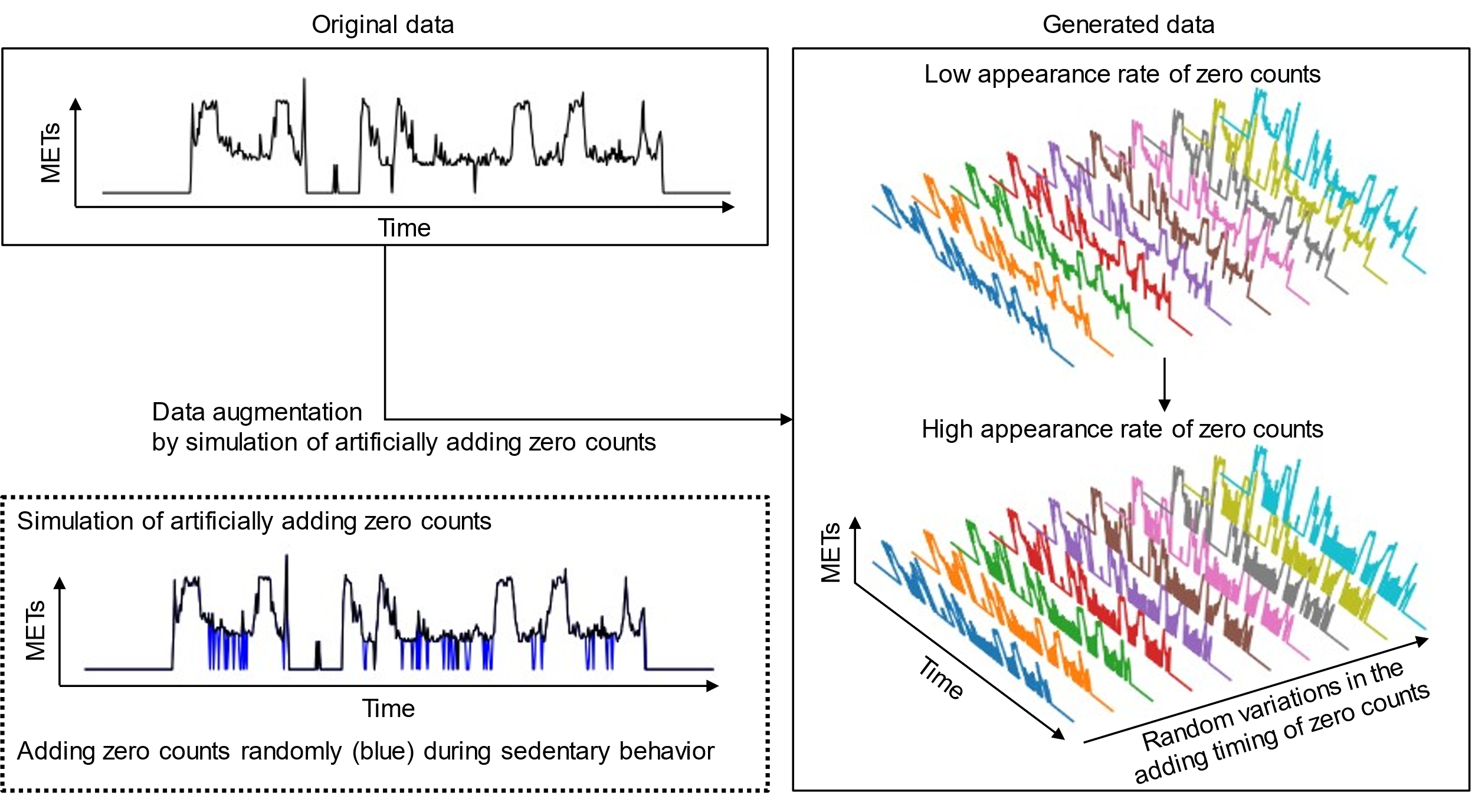


Simulation for augmenting the variety of data from the original data of participants. Zero counts were artificially added randomly during the sedentary behavior according to the probability of appearance of zero counts ($r_{sedentary}^{zero}$). $r_{sedentary}^{zero}$ was gradually increased, and ten repetitive simulations were performed at each $r_{sedentary}^{zero}$.
